# Clinical outcomes of salvage radiotherapy in patients with supraclavicular lymph node metastases after esophagectomy

**DOI:** 10.3389/fonc.2022.1016150

**Published:** 2023-01-11

**Authors:** Zhang Ping, Zhen Chanjun, Bai Wenwen, Chen Mingyue, Su Quanbing, Wang Yajing, Zhou Zhiguo

**Affiliations:** Department of Radiation Oncology, the Fourth Hospital of Hebei Medical University, Shijiazhuang, China

**Keywords:** esophageal cancer, squamous cell carcinoma, supraclavicular lymph node metastases, salvage radiotherapy, esophagectomy

## Abstract

**Purpose:**

To evaluate the clinical outcomes of salvage radiotherapy in patients with supraclavicular lymph node (SCLN) metastases after esophagectomy.

**Methods:**

After initial esophagectomy (R0 resection), clinical outcomes in patients with esophageal squamous cell carcinoma with SCLN metastases during follow-up were retrospectively analyzed.

**Results:**

A total of 114 patients were split into two groups: the salvage radiotherapy (SR) (n=89) and the control (NSR) (without salvage radiotherapy, n=25). The overall survival rates of 1 year, 3 years and 5 years were 81.6%, 31.4% and 8.6%, accordingly. The 1-year and 3-year survival after SCLN metastases (SASM) rates were 40.2% and 14.5%, respectively; the median SASM time was 10 months. In the SR group, the SASM rates of 1-year and 3-year were 48.1% and 18.9%, compared to 12.0% and 0% in the NSR group (*p*<0.001). Patients in the SR group who received combined radiochemotherapy experienced 1-year and 3-year SASM rates of 62.6% and 33.4%, compared to 41.9% and 16.5% with single radiotherapy (*p*<0.001). The salvage radiation dose revealed that the 1-year and 3-year SASM rates turned out to be 56.5% and 23.4% in group of ≥60 Gy, and 29.2% and 7.5% in group of <60 Gy (*p*<0.001). According to multivariate analysis, combined visceral metastases (CVM), combined mediastinal failure (CMF), salvage radiotherapy, salvage radiation dose and salvage treatment method possibly were identified as important prognostic variables. After propensity score matching (PSM), the above results were similar to those before PSM, except for that only salvage radiotherapy is possibly independent prognostic variables for survival after SCLN metastases in multivariate analysis.

**Conclusion:**

It is possible that **s**alvage radiotherapy can increase the survival rate of patients who receive SCLN metastases following esophagectomy.

## Introduction

In the seventh edition of the American Joint Committee on Cancer staging system for esophageal squamous cell carcinoma in 2010, celiac axis nodes and paraesophageal lymph nodes in the neck are categorized as regional lymph node metastases instead of distant metastatic spread. However, supraclavicular lymph nodes (SCLN) remain as distant metastases regardless of where the primary tumor is located (1, 2). According to reports, for patients having esophageal cancers, the incident rate of SCLN metastases turned out to be about 10% (3, 4). However, the treatment strategy for SCLN metastases with esophageal cancer (EC) is still controversial. Some studies showed that SCLN dissection could provide a better chance of survival (5–8). On the other hand, Shim et al. (9) demonstrated that the SCLN dissection could not lead to a survival benefit.

After radical esophagectomy, the metastasis rates of SCLN with EC ranged from 10% to 40% in some reports (3, 4, 10). The survival rate of 5-year reached about 25% when it came to patients receiving SCLN metastases after esophagectomy (4, 7, 11). Once SCLN metastasis occurred after esophagectomy, the prognosis was worse. Even though perioperative chemotherapy or chemoradiotherapy prior to or after surgery can be a standard treatment for advanced resectable EC, the therapeutic value of adding radiotherapy in thoracic EC with SCLN metastases determined by pre-treatment imaging to be resectable is still debatable. Prognosis ought to be considered while deciding on a treatment approach. This research was conducted to elucidate the survival results for patients receiving SCLN metastases after esophagectomy treated with salvage radiotherapy.

## Patients and methods

### Patient selection

Between January 2006 and December 2012, a retrospective analysis was performed on 114 patients with resectable EC who were diagnosed as SCLN metastases after radical resection. The following were the criteria for inclusion: 1) patients with EC who had received initial esophagectomy (R0 resection); 2) the thoracic esophagus housed the primary tumor; 3) patients with pathologically confirmed squamous cell carcinoma and without SCLN metastases or distant metastases before surgery; 4) patients with SCLN metastases confirmed squamous cell carcinoma by pathological analysis after surgery; 5) patients without salvage resection treatment after SCLN metastases; 6) patients with no other major medical diseases and second primary tumor except for EC.

All patients were placed into two categories: the salvage radiotherapy (SR) group receiving radiotherapy to SCLN with/without chemotherapy, n=89 (78.1%); the control (NSR) group without salvage radiotherapy, n=25 (21.9%). According to whether visceral metastases or mediastinal failure occurred when SCLN metastases were confirmed, the patients were further divided into the combined visceral metastases (CVM) group and non-combined visceral metastases (NCVM) group, or combined mediastinal failure (CMF) group and non-combined mediastinal failure (NCMF) group. According to the failure model at the time supraclavicular nodes metastases proved, all patients were deeply divided into four groups: single SCLN metastases (SSM), SCLN and visceral metastases (SVM), SCLN metastases and mediastinal failure (SMMF), SCLN and visceral metastases and mediastinal failure (SVMMF).

### Treatment scheme

Radical resection included right transthoracic esophagectomy and mediastinal dissection with extensive lymphadenectomy. After SCLN metastasis, patients of the SR group underwent three-dimensional conformal radiotherapy (3D-CRT) plus electron irradiation or an intensity-modulated radiotherapy (IMRT). If patients had combined mediastinal failure, radiation fields included the mediastinal recurrence region. The involved radiation field was adopted in patients of the SR group. Salvage radiotherapy was performed accompanied by a median dose of 54 Gy (18~66 Gy), 1.8~2.0 Gy per fraction, 5 times a week. The highest dose below 45 Gy was used to represent the radiation dose limit for the spinal cord. The average dose and V20 for the lungs were constrained to 20 Gy and 30% separately.

### Follow-up

Follow-up was carried out every three months for the first year after surgery and each six to twelve months after that. Every year for the first five years, as well as when there were clinical indications, CT scans and esophagogastroscopy assessments would be conducted. Once SCLN metastasis was confirmed, a systemic examination was required to reassess the clinical stage. Then, the follow-up time was changed per 1-2 months for the first 1 year and 3-6 months for subsequent years. The average duration of follow-up was 25 months.

### Statistical analysis

The whole statistical studies were carried out employing SPSS 18.0 software (SPSS Inc.). The Pearson chi-square test was used to compare the categorical variables among groups. The overall survival (OS) was computed from the time of surgery to the time of any event of death or the final follow-up. The non-SCLN metastases survival (NSMS) was calculated from the date of surgery to the date of SCLN metastases confirmed. The survival after SCLN metastases (SASM) was computed from the day that SCLN metastases were confirmed until the date of any event of death or the last follow-up. The survival curves were plotted to adopt the Kaplan-Meier method and compared by the log-rank test. Multivariate analysis was performed using the cox proportional hazards model. Propensity score matching (PSM) was employed to reduce the bias from baseline confounding variables. Every statistical test was two-sided with significance defined as *p<*0.05.

## Results

### Patient selection

Patient features are listed in **Table 1**. A total of 114 patients would be separated into the salvage radiotherapy (SR) group (n=89) and the control (NSR) group (without salvage radiotherapy, n=25).

**Table 1:** Patient characteristics before and after PSM.

Parameters	Before PSM		After PSM	
	SR group (n=89)	NSR group (n=25)	SR group (n=25)	NSR group (n=25)
Gender, n (%)				
Male	67 (75.3%)	20 (80.0%)	8 (32.0%)	20 (80.0%)
Female	22 (24.7%)	5 (20.0%)	17 (68.0%)	5 (20.0%)
Median age (range), years				
≤60 years, n (%)	54 (60.7%)	14 (56.0%)	19 (76.0%)	14 (56.0%)
>60 years, n (%)	35 (39.3%)	11 (44.0%)	6 (24.0%)	11 (44.0%)
Lenth, cm	5.1±2.1	5.2±1.7	5.1±1.5	5.2±1.7
Tumor location, n (%)				
Upper	16 (18.0%)	5 (20.0%)	9 (36.0%)	5 (20.0%)
Middle	65 (73.0%)	16 (64.0%)	16 (64.0%)	16 (64.0%)
Lower	8 (9.0%)	4 (16.0%)	0 (0%)	4 (16.0%)
T stage				
T1	1 (1.1%)	0 (0%)	0 (0%)	0 (0%)
T2	20 (22.5%)	2 (8.0%)	5 (20.0%)	2 (8.0%)
T3	66 (74.2%)	22 (88.0%)	19 (76.0%)	22 (88.0%)
T4	2 (2.2%)	1 (4.0%)	1 (4.0%)	1 (4.0%)
N stage				
N0	28 (31.5%)	10 (40.0%)	5 (20.0%)	10 (40.0%)
N1	32 (36.0%)	12 (48.0%)	11 (44.0%)	12 (48.0%)
N2	26 (29.2%)	3 (12.0%)	9 (36.0%)	3 (12.0%)
N3	3 (3.4%)	0 (0.0%)	0 (0%)	0 (0.0%)
TNM stage				
II	22 (24.7%)	6 (24.0%)	6 (24.0%)	6 (24.0%)
III	40 (44.9%)	12 (48.0%)	12 (48.0%)	12 (48.0%)
IV	27 (30.3%)	7 (28.0%)	7 (28.0%)	7 (28.0%)
CVM				
No	62 (69.7%)	18 (72.0%)	18 (72.0%)	18 (72.0%)
Yes	27 (30.3%)	7 (28.0%)	7 (28.0%)	7 (28.0%)
CMF				
No	44 (49.4%)	13 (52.0%)	12 (48.0%)	13 (52.0%)
Yes	45 (50.6%)	12 (48.0%)	13 (52.0%)	12 (48.0%)
Salvage radiation dose				
<60 Gy	27 (30.3%)	0 (0.0%)	5 (20.0%)	0 (0.0%)
≥60 Gy	62 (69.7%)	0 (0.0%)	20 (80.0%)	0 (0.0%)
Salvage treatment methods				
Radiochemotherapy	27 (30.3%)	0 (0.0%)	6 (24.0%)	0 (0.0%)
Radiotherapy	62 (69.7%)	0 (0.0%)	19 (76.0%)	0 (0.0%)
Support care	0 (0.0%)	17 (68.0%)	0 (0.0%)	17 (68.0%)
Chemotherapy	0 (0.0%)	8 (32.0%)	0 (0.0%)	8 (32.0%)

### Influences of salvage radiotherapy on survival

**Figure 1:**
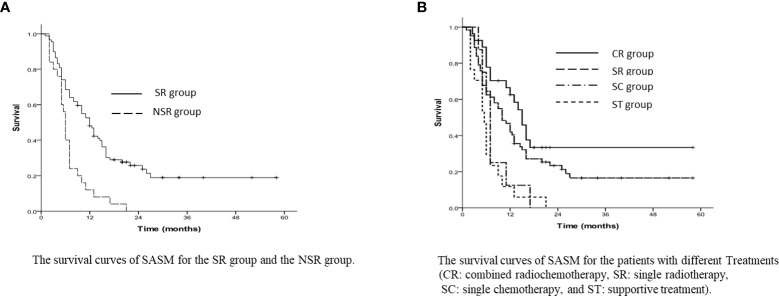
**(A)** The survival curves of SASM for the SR group and the NSR group. **(B)** The survival curves of SASM for the patients with different Treatments (CR, comt ed radiochemotherapy; SR, single radiotherapy; SC, single chemotherapy; and ST, supportive treatment).

The average survival duration of whole patients was 24 months (range 5~67 months), and the 1-year, 3-year and 5-year OS rates turned out to be 81.6%, 31.4% and 8.6%, separately. The NSMS rates for 1-year, 3-year and 5-year were 42.5%, 9.7% and 0%, separately, and the average NSMS time was 11 months (range 1~54 months). In all patients, the 1-year and 3-year SASM rates were 40.2% and 14.5%, respectively; the median SASM time was 10 months (range 1~58 months). The SR group had 1-year and 3-year SASM rates of 48.1% and 18.9%, compared to NSR group’s 12.0% and 0%; for the SR group and the NSR group, the median SASM duration reached 12 months (range 9.6~14.4 months) and 6 months (range 5.4~6.6 months), respectively (*p*<0.001, **Figure 1A**).

As for the SR group, patients receiving combined radiochemotherapy had 1-year and 3-year SASM rates of 62.6% and 33.4%, and with single radiotherapy were 41.9% and 16.5%, respectively. However, they were longer than those in the patients with single chemotherapy (25% and 0%) and with the best supportive treatment (11.8% and 0%) (*p*<0.001, **Figure 1B**).

### Influences of tumor metastases and mediastinal failure on survival

**Figure 2:**
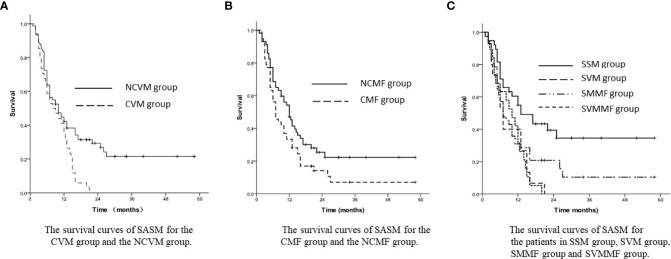
**(A)** The survival curves of SASM for the CVM group and the NCVM group. **(B)** The survival curves of SASM for the CMF group and the NCMF group. **(C)** The survival curves of SASM for the patients in SSM group, SVM group, SMMF group and SVMMF group.

In the subgroup analysis, the patients’ 1-year and 3-year SASM rates turned out to be 35.3% and 0% in the CVM group, while 42.3% and 21.5% in the NCVM group, respectively (*p*=0.004, **Figure 2A**). Patients in the NCMF group had a greater 3-year SASM rate (22.2%) than those in the CMF group (7.0%) (*p*=0.041, **Figure 2B**).

In the stratified analysis, the 1-year and 3-year SASM rates were 54.9% and 34.5% in SSM group, 31.9% and 0% in SVM group, 31.0% and 10.4% in SMMF group, and 40.0% and 0% in SVMMF group (*p*=0.003, **Figure 2C**).

**Figure 3:**
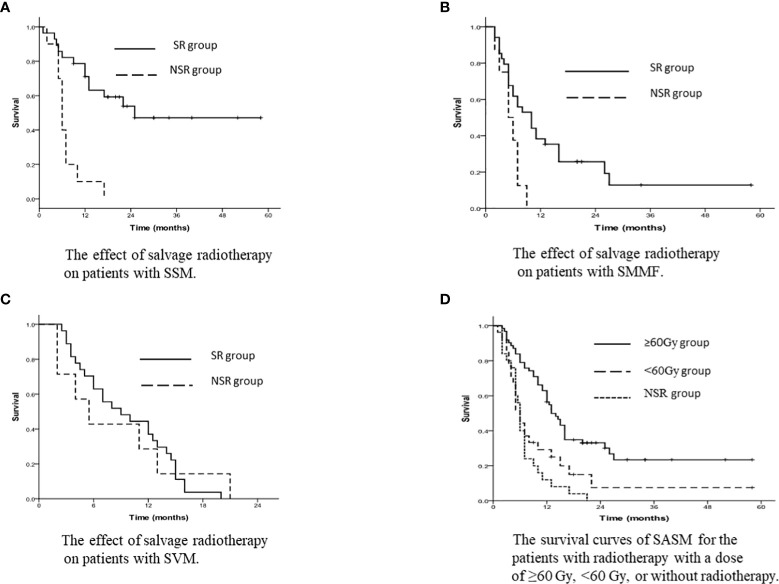
**(A)** The effect of salvage radiotherapy on patients with SSM. **(B)** The effect of salvage radiotherapy on patients with SMMF. **(C)** The effect of salvage radiotherapy on patients with SVM. **(D)** The survival curves of SASM for the patients with radiotherapy with a dose of ≥60 Gy, <60 Gy, or without radiotherapy.

According to the subgroups of salvage treatment, the 1-year and 3-year SASM rates for SSM patients in the SR group were 71.1% and 47.1%, and in the NSR group were 10.0% and 0% (p<0.001, **Figure 3A**).

For SMMF patients, the 1-year and 3-year SASM rates were 38.2% and 12.8% in the SR group, and 0% and 0% in the NSR group (*p*=0.012, **Figure 3B**).

The 1-year and 3-year SASM rates for the patients with SVM were 37.0% and 0% in the SR group, while 28.6% and 0% in the NSR group (*p*=0.003, **Figure 3C**).

### Influences of radiation dose on survival

According to the salvage radiation dose, the 1-year and 3-year SASM rates were 56.5% and 23.4% for patients receiving radiotherapy with a dose of ≥60 Gy, and 29.2% and 7.5% in patients receiving <60 Gy radiotherapy, respectively (p<0.001, **Figure 3D**).

### Multivariate analysis of prognostic factors

A multivariate analysis showed that combined visceral metastases, combined mediastinal failure, salvage radiotherapy, salvage radiation dose and salvage treatment method are possibly independent prognostic variables for overall survival (**Table 2**). 92 patients passed away up to the end day of follow-up. The most causes of death were local failure and distant metastasis shown in **Table 3**.

**Table 2:** Univariate and multivariate analyses of prognostic factors before PSM.

Factors		Univariate analysis			Multivariate analysis		
		1-year Survival	3-year Survival	*p* value	Risk ratio	95% CI	*p* value
Sex	Male	37.7%	8.6%	0.066	1.192	0.659-2.155	0.562
	Female	48.1%	28.3%				
Age	≤60y	46.9%	17.1%	0.477	0.802	0.500-1.287	0.361
	>60y	30.4%	9.6%				
Tumor location	Upper	38.1%	19.0%	0.825	1.204	0.536-2.705	0.653
	Middle	41.7%	14.1%				
	Lower	33.3%	16.7%				
SCLN position	Left side	39.2%	9.8%	0.198	0.700	0.363-1.352	0.289
	Right side	45.6%	20.6%				
	Both sides	23.5%	11.8%				
CVM	No	42.3%	21.5%	0.004	2.127	1.279-3.538	0.004
	Yes	35.5%	0%				
CMF	No	47.0%	22.2%	0.041	0.638	0.413-0.985	0.043
	Yes	33.3%	7.0%				
Salvage radiation	Yes	48.1%	18.9%	<0.000	4.326	1.713-10.922	0.002
	No	12.0%	0%				
Salvage radiation dose	<60 Gy	29.2%	7.5%	<0.000	2.877	1.649-5.019	<0.000
	≥60 Gy	56.5%	23.4%				
	No	12.0%	0%				
Salvage treatment methods	Radiochemotherapy	62.5%	33.4%	<0.000	2.079	1.164-3.711	0.013
	Radiotherapy	41.9%	16.5%				
	Supportive treatment	11.8%	0%				
	chemotherapy	12.5%	0%				

**Table 3:** The causes of death for patients with SCLN metastases after esophagectomy.

Death causes	N	Proportion (%)
Local failure	49	53.3
Distant metastases	16	17.4
Local failure + distant metastases	19	20.6
Perforation/Blooding	3	3.3
Others	5	5.4
Total	92	100

### Results analysis after PSM

**Figure 4:**
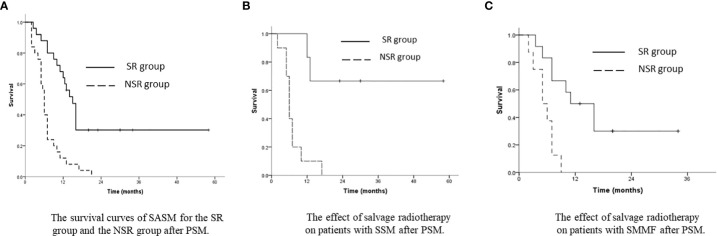
**(A)** The survival curves of SASM for the SR group and the NSR group after PSM. **(B)** The effect of salvage radiotherapy on patients with SSM after PSM. **(C)** The effect of salvage radiotherapy on patients with SMMF after PSM.

**Figure 5:**
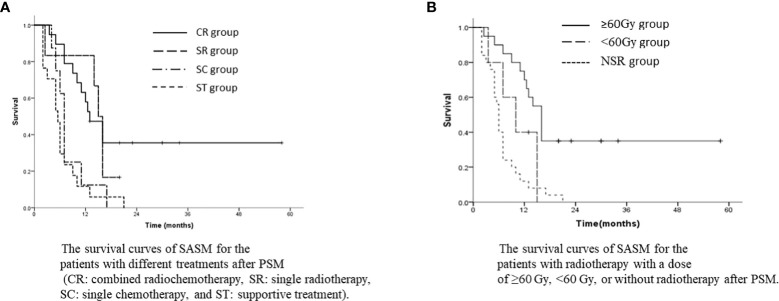
**(A)** The survival curves of SASM for the patients with different treatments after PSM (CR, combined radiochemotherapy; SR, single radiotherapy; SC, single chemotherapy; and ST, supportive treatment). **(B)** The survival curves of SASM for the patients with radiotherapy with a dose of ≥60 Gy, <60 Gy, or without radiotherapy after PSM.

After PSM, the SASM rate of the SR group was better than that of the NSR group. The 1-year and 3-year SASM rates were 64.0% and 30.2% in the SR group, and 12.0% and 0% in the NSR group (p<0.001, **Figure 4A**). For patients with SSM and SMMF, radiotherapy was superior to without radiotherapy (**Figures 4B, C**). Meanwhile, the SASM rates of the patients received radiochemotherapy were significantly higher than those with single chemotherapy and with the best supportive treatment (p<0.001, **Figure 5A**). According to the salvage radiation dose, the patients received a dose of ≥60Gy have a better survival than those with <60Gy (p<0.001, **Figure 5B**). The above results were similar to those before PSM. However, no statistical significance was found in the prognosis of patients with SCLN metastases combined with different metastatic sites. For the patients with SVM, no statistical difference was found in whether they received radiotherapy (p=0.430). A multivariate analysis showed that only salvage radiotherapy is possibly independent prognostic variables for SASM after PSM (**Table 4**).

**Table 4:** Univariate and multivariate analyses of prognostic factors after PSM.

Factors		Univariate analysis			Multivariate analysis		
		1-year Survival	3-year Survival	*p* value	Risk ratio	95% CI	*p* value
Sex	Male	32.1%	5.4%	0.072	0.600	0.232-1.551	0.292
	Female	45.5%	25.6%				
Age	≤60y	45.5%	15.8%	0.300	0.785	0.367-1.679	0.533
	>60y	23.5%	11.8%				
Tumor location	Upper	35.5%	14.3%	0.252	1.383	0.357-5.361	0.639
	Middle	43.8%	16.5%				
	Lower	0%	0%				
SCLN position	Left side	28.6%	0%	0.180	0.693	0.224-2.144	0.525
	Right side	50.0%	19.6%				
	Both sides	12.5%	12.5%				
CVM	No	33.0%	21.4%	0.378	1.228	0.514-2.931	0.644
	Yes	50.0%	0%				
CMF	No	40.0%	16.0%	0.858	1.008	0.504-2.014	0.982
	Yes	36.0%	9.1%				
Salvage radiation	Yes	64.0%	30.2%	<0.000	3.937	1.040-14.923	0.044
	No	12.0%	0%				
Salvage radiation dose	<60 Gy	40.0%	0%	<0.000	2.691	0.817-8.860	0.104
	≥60 Gy	70.0%	30.5%				
	No	12.0%	0%				
Salvage treatment methods	Radiochemotherapy	83.3%	16.7%	<0.000	1.528	0.607-3.846	0.368
	Radiotherapy	57.9%	35.5%				
	Supportive treatment	11.8%	0%				
	chomotherapy	12.5%	0%				

### Toxicity and side effects

No grade 5 toxicities were observed in patients who could be followed up. The main radiation-related side effects were radiation esophagitis and radiation pneumonia in the SR group. The incidence of radiation esophagitis (≤grade 2) was 78.7%, and only 6 patients (6.7%) were observed grade 3 or above. For radiation pneumonia no grade 3 or above was observed. No patients with radiation myelitis were observed. The incidence of radiation dermatitis (≤grade 2) was 58.4%, and only 13 patients (14.6%) were observed grade 3 or above. 81 patients were recorded as myelosuppression, 13 patients (14.6%) were observed grade 3 and above. Radiation-related side effects were shown in **Table 5**.

**Table 5:** Radiation-related side effects.

Group	Grade	Radiation esophagitis (%)	Radiation pneumonia (%)	radiation dermatitis (%)	myelosuppression (%)
SR	≤2	70 (78.7)	76 (85.4)	52 (58.4)	68 (76.4)
	≥3	6 (6.7)	0 (0)	13 (14.6)	13 (14.6)
	miss	13 (14.6)	13 (14.6)	24 (27.0)	8 (9.0)

## Discussion

There is still controversy about the SCLN metastases of esophageal cancer as distant metastases, even though SCLN metastases is not included in regional lymph nodes in the UICC/AJCC TNM classification. Most clinicians believe that the presence of M1 disease precludes curative treatment and the prognosis of patients with SCLN metastases is poor. However, there were few reports on the impact of lymph node metastases, focusing on the influence of SCLN metastases on survival (12). Especially, there were less reports about the salvage treatment for SCLN metastases.

Some studies showed that although the prognosis was poor, patients with cervical lymph node recurrence following curative resection still had a chance to be cured (13, 14). In our study, SCLN metastases were observed in nearly 45% of patients after surgery within one year. About 90% of patients developed SCLN metastases within 3 years after surgery. The postoperative unilateral SCLN metastasis rate was higher than bilateral SCLN metastasis rate, and the metastasis rate of right supraclavicular area was higher than that of left supraclavicular area. Similar results were reported in Li’s research (10).

At present, there is not established standard of treatment for esophageal cancer patients with SCLN metastasis after surgery, but systemic chemotherapy has been widely accepted as the standard treatment for patients with distant organ metastases. In our study, the advantages in patients receiving salvage radiotherapy after SCLN metastases were confirmed. The 3-year survival rate of patients with salvage radiotherapy was 18.9%, and the patients without salvage radiotherapy died in 3 years. In particular, it was showed that salvage radiochemotherpy was effective for the survival of these patients. Lu et al. (15) reported that the median survival time of patients with SCLN metastasis after surgery receiving radiotherapy or radiochemotherapy was 18 months, and radiochemotherapy should be considered an effective treatment for patients with lymph node recurrence including SCLN after radical resection of thoracic esophageal squamous cell carcinoma. However, only 19 patients with postoperative SCLN recurrence were reported, and there was no information on the comparison of radiotherapy with radiochemotherapy.

Nowadays, the presence of SCLN metastasis, similar to visceral metastasis, may be considered a contraindication for curative treatment. Chen et al. (16) showed that multimodality treatment may also improve the survival rate of patients with distant organ metastasis, but chemotherapy alone has not been identified as a favorable prognostic factor. In our study, there was similar results that multimodality salvage treatment of patients with SCLN metastasis can improve the survival rate than chemotherapy alone or supportive treatment.

The prognoses of patients with salvage radiotherapy were different between single SCLN metastasis and multiposition failure, especially visceral metastasis. Our results showed that the survival rate of SSM group was higher than that of patients with more than one position recurrence. Watanabe et al. (14) also found similar results, that is, patients with isolated cervical lymph node recurrence had a longer survival time from diagnosis of recurrence than patients with recurrence of other sites. Chen et al. (16) also demonstrated that the presence of single metastasis seemed to be beneficial to overall survival compared to multiple metastases in patients with esophageal cancer.

The 3-year survival rate of patients with SSM after salvage treatment was 34.5%. However, those patients with distant organ metastasis all died within the following three years regardless of salvage treatment. Therefore, according to our results, it is recommended that patients with only SCLN metastasis after radical resection should be treated with salvage radiotherapy.

It was reported that the prognosis of patients with local recurrence after esophagectomy receiving a radiation dose of nearly 60 Gy was good (17, 18). In our study, we also found that the survival time of patients receiving salvage radiation dose ≥60 Gy was longer than that of patients with radiation dose <60 Gy and without salvage radiotherapy. The reason may be that higher dose may lead to more local control rates and longer survival time.

The causes of death in most patients were local failure (53.3%) and distant metastasis (38%). According to our results, most patients with SCLN recurrence after surgery also should be concerned about the treatment of local failure.

Salvage radiotherapy may improve the survival rate of patients with SCLN metastases after esophagectomy. Combined radiochemotherapy, non-combined visceral metastases, non-combined mediastinal failure, and salvage radiation dose ≥60 Gy were associated with better prognosis for patients with SCLN metastases after esophagectomy.

## Data availability statement

The original contributions presented in the study are included in the article/supplementary material. Further inquiries can be directed to the corresponding author.

## Ethics statement

The studies involving human participants were reviewed and approved by Ethics Committee of Drug Clinical Trials, the Fourth Hospital of Hebei Medical University. The patients/participants provided their written informed consent to participate in this study.

## Author contributions

All authors made substantial contributions to conception and design, acquisition of data, or analysis and interpretation of data; took part in drafting the article or revising it critically for important intellectual content; gave final approval of the version to be published; and agree to be accountable for all aspects of the work.
